# Helmet Use Amongst Equestrians: Harnessing Social and Attitudinal Factors Revealed in Online Forums

**DOI:** 10.3390/ani5030373

**Published:** 2015-07-17

**Authors:** Laura Haigh, Kirrilly Thompson

**Affiliations:** The Appleton Institute, Central Queensland University, 44 Greenhill Road, Wayville, SA 5034, Australia; E-Mail: kirrilly.thompson@cqu.edu.au

**Keywords:** equestrian, horse, injury, helmet, safety, risk, online forum, barriers, enablers, behavior change, injury prevention

## Abstract

**Simple Summary:**

Epidemiological research details a high rate of horse-related injury, despite technical countermeasures being widely available and largely affordable. Whilst barriers to engaging in preventative behavior such as helmet-use have been identified, less attention has been given to enabling factors. These factors could contribute to the design of more effective injury prevention interventions. To identify barriers as well as enablers in an Australian context, we explored how riders discussed helmet use amongst one another in an online setting. Our analysis revealed that social relations heavily influenced safety behavior. In particular, we identified three attitudes that affected helmet use: “I Can Control Risk”, “It Does Not Feel Right” and “Accidents Happen”.

**Abstract:**

Equestrian activities pose significant head injury risks to participants. Yet, helmet use is not mandatory in Australia outside of selected competitions. Awareness of technical countermeasures and the dangers of equestrian activities has not resulted in widespread adoption of simple precautionary behaviors like helmet use. Until the use of helmets whilst riding horses is legislated in Australia, there is an urgent need to improve voluntary use. To design effective injury prevention interventions, the factors affecting helmet use must first be understood. To add to current understandings of these factors, we examined the ways horse riders discussed helmet use by analyzing 103 posts on two helmet use related threads from two different Australian equestrian forums. We found evidence of social influence on helmet use behaviors as well as three attitudes that contributed towards stated helmet use that we termed: “I Can Control Risk”, “It Does Not Feel Right” and “Accidents Happen”. Whilst we confirm barriers identified in previous literature, we also identify their ability to support helmet use. This suggests challenging but potentially useful complexity in the relationship between risk perception, protective knowledge, attitudes, decision-making and behavior. Whilst this complexity is largely due to the involvement of interspecies relationships through which safety, risk and trust are distributed; our findings about harnessing the potential of barriers could be extended to other high risk activities.

## 1. Introduction

Involvement in sport and physical recreation has a positive impact on health and quality of life, providing socio-economic benefits to wider public health [1]. Despite this, the potential risk and occurrence of injury associated with physical activity can act as a barrier to continued participation [1]. This has prompted researchers to advocate for the incorporation of injury prevention strategies into sport promotion [1,2,3,4]. Horse riding and related activities are particularly dangerous [5,6,7,8]. Equestrian activities pose injury risks to an estimated quarter of a million Australians [9]. “The rider is unrestrained and is travelling on a largely unpredictable animal capable of speeds of up to 70 kph and of kicking with a force of up to 1 ton” [5] (p. 5). Not only are horses large, fast and strong, they are decision-making animals of prey with a heightened flight response [10].

There is a sophisticated body of work documenting injury statistics, identifying horse-related injury risk factors and determining high-risk groups [5,8,11,12,13,14,15,16,17,18]. Both Australian and International research into horse related injury highlight the importance of encouraging the use of safety standard helmets to be consistently adopted by riders [19,20,21,22]. This is further demonstrated by research that charts the positive impact of safety helmet use [23,24]. Indeed, Safe Work Australia [25] advocate the use of Australian standard safety helmets alongside the development of skills and confidence in horse interaction. Yet, despite awareness of the critical importance of helmet use in preventing head injury, adoption by equestrians still lacks consistency [11,19,26,27,28]. Until the use of helmets whilst riding horses is legislated in Australia, there is an urgent need to improve voluntary use.

Recommendations to increase helmet use and improve overall safety typically include training, education, and awareness-raising activities such as wider dissemination of injury statistics [12,19,25,27]. However, increasing the levels of risk literacy amongst equestrians may not necessarily lead to safer equestrian practices [29]. This is largely because the ways in which people perceive and respond to risk are not straightforward. They are subject to psychological and cultural contextualization [30], as are the values, attitudes, beliefs and practices of equestrians towards issues of horse-related risk and safety. As such, equestrian injury statistics and risk factors can be considered a product of individual, social and cultural attitudes and behaviors.

Unlike helmet use amongst cyclists and motorcyclists on Australian public roads [31,32], helmet use is not mandatory for horse riders. Helmet use amongst equestrians therefore provides an illuminating case study of voluntary helmet use. Whilst voluntary helmet use has been discussed in detail in relation to cycling [33,34], motorcycling [35,36] and alpine sports [37,38,39], those activities do not involve human interaction with another sentient, decision-making being such as the horse [10]. Indeed, previous research on human and horse relations demonstrates the ways in which riders’ risk and safety decisions depend on how they perceive the quality of their human–horse relationship [40,41].

Moreover, there is a particular need to understand and address the ways in which equestrians perceive, respond to and talk about risk and safety amongst themselves. These can be inferred from “everyday talk” [42] in discussions and conversations where beliefs and behaviors are often advocated, justified and challenged. For example previous research [28,29] demonstrates the impact that negative attitudes, altered risk perception and physical discomfort has had on the inconsistent uptake of helmet use. As that research was biased towards barriers, there is a need to identify enablers and consider their value in behavior change campaigns.

## 2. Method

To identify attitudes and beliefs surrounding helmet use, we analyzed two public-access forums on Australian equestrian websites. This provided an unobtrusive means of exploring the “everyday talk” [42] of equestrians, driven by their own concerns. The construction of forum-as-field-site has been successfully utilized by health researchers looking into discourses surrounding topics such as breastfeeding, early onset dementia and disordered eating habits [42,43,44].

A sampling strategy was devised to select equestrian-based discussion forums, following Callaghan and Lazard [42]. An Internet search was conducted using the terms “Australia”, “Forum” and “Horse” or “Equestrian”. The inclusion criterion for forum selection was: high variety of topics; high level of posting activity; ability to search the forums; publically accessible content and, containing a “.au” identifier. The two forums selected were accessed publicly, without the need for payment or membership. They contained a broad variety of topics (from horse health to training) but also had differing target audiences (one was noted to be a “junior rider” forum while the other was a general equestrian discussion forum). The forums were initially searched using terms: “Helmets” and “Helmet use”. Inclusion criteria for threads included: relevance of the topic to the research aims; high level of response/posting activity and variance of posters. Both threads commenced with a forum member enquiring about, or commenting on, the helmet wearing behaviors of fellow forum members. Thread 1 contained 50 posts from 37 members, with discussion taking place within the time span of a month in 2013. Thread 2 contained 53 posts from 43 members, taking place over a weeklong period in 2004. Neither of the authors were involved in thread instigation or participation.

Data collection followed a protocol used by Rodriquez [43], who only analyzed posts from publically published and accessible forum posts, and devised pseudonyms for the already generally anonymous posters’ nicknames. This study was approved by the CQUni Human Research Ethics Committee.

The method can be summarized in the flowchart below:

**Flow Chart 1:**
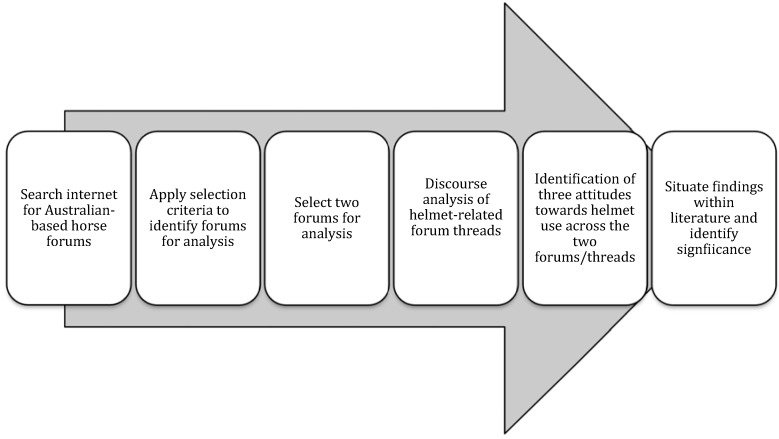
Six steps comprising the research methodology.

Discourse analysis enabled the identification of factors affecting helmet use. The 103 separate posts were analyzed. First, they were read and re-read before being inductively analyzed as whole statements. Each post, or “statement”, was broken down and analyzed sentence by sentence with particular focus on discursive attitudes expressed towards helmet use. Following Green and colleagues [45], relationships between these codes were explored. Patterns between factors, risk perception and different stated safety behaviors were documented, as were differing values associated with various equestrian activities within the posts. To identify attitudes or factors that enabled helmet use, we focused attention on discourse surrounding stated consistent helmet use or stated opposition to non-use. Analysis was conducted primarily by Haigh in conjunction with Thompson whose personal involvement in equestrian activities allowed for a nuanced interpretation of horse-specific terminology and concepts.

## 3. Results

In the “everyday talk” [42] of forum users, we identified numerous social and attitudinal factors significantly affecting helmet use, risk perception and injury mitigation behaviors. Overall, they were deeply entrenched in the social relations of their “eque-cultures” [46]. Three attitudes that affected helmet use have been selected for discussion in this paper, based on their overarching representation across both forums:

(1) “I Can Control Risk”;
(2) “It Does Not Feel Right”;
(3) “Accidents Happen”.

### 3.1. The Impact of Social Relations on Helmet Use

In forum threads, what was often perceived as a “personal” choice to wear or not wear a helmet was socially influenced. Indeed, a large number of forum posters justified their helmet (non)use through stories of peer behaviors. Social influence was seen to act negatively on some forumites, who both explicitly and implicitly linked their helmet use to the riding culture or social environment they were within. For example:

- One rider openly claimed to be inconsistent with helmets, asserted this as a personal choice, but justified their actions with the claim that: “*I know people that don’t even own one*”.
- A decision to not wear a helmet was stated as supported by family.
- The influence of experienced riding peers altered the helmet use of one rider, who noted that “*I spent a few months training with a well-known showjumper in NSW, basically nobody wears a helmet there and I just got used to it*”. This same rider made an exception and noted that they would wear a helmet “*If kids are watching*”.

This last example highlights that while social influence and peer behavior operated on occasion as a negative force on helmet use, it also had the potential to enable and support helmet use. This can be seen in the following examples:

- One rider justified their helmet use by suggesting that non-helmeted riders “*are being a bit selfish. Not because they are doing a sport where there are safety measures that may limit the damage done but because they aren’t considering their families*”.
- Another rider stated that being injured while not wearing a helmet “*is not fair to those left behind*”.
- Helmet use was positioned as something you do for “*those who love you*”.
- One rider linked their helmet use to being raised in a family involved in racing (Racing Australia [47] mandates helmet use at any competition).

In these examples, attitudes and behaviors towards helmet use were overtly influenced by perceived social responsibility and “care” for some riders, and more subtly formed by social influences and norms for other riders. This gave rise to a judgmental rhetoric. Alongside this pervasive social dimension, three attitudes were found to influence helmet use: “I Can Control Risk”, “It Does Not Feel Right” and “Accidents Happen”. These attitudes are outlined below.

### 3.2. Attitudes Influencing Helmet Use

#### 3.2.1. “I Can Control Risk”

Most riders were generally positive about helmet use. Despite this, when an attitude of “I Can Control Risk” was present, the necessity of helmet use was affected by varying risk mitigation strategies. The extent of risk was perceived as depending on the type of riding activity, the age and training of the horse and importantly, the relationship between horse and rider. Risk mitigation strategies available to these riders included: the rider’s own skill set and experience; knowledge of horse behavior; strength of horse and human relationship, and helmet use. Interplay between these factors affected helmet use even for those who stated to be generally consistent in wearing. For example:

- One rider noted: *I very rarely ride without one. In fact the only time I ride without one, is when I bring them up from the paddock or I ride* (my own horse-name removed) *tackless* (without a saddle and bridle) *for a few minutes’*.
- Another discussed riding helmetless on a few occasions while bringing the horse they trust “110%” up from their own paddock. This rider also noted that they would only ever walk their horse up (travelling at a slower and safer pace) and would not risk it if their horse were having a “*hyper*” day.
- Another suggested that the “*1%*” of the time they would not ride with a helmet would only be when “*jumping on*” their old horse to bring them up to their gate.
- The attitude that “safer” situations could potentially negate the need to wear a helmet was even held by one member who claimed quite strongly that riding without a helmet “*made zero sense*”, as it was later noted that the reason why they did not ride without a helmet was they knew their horse to be “*unpredictable*”.

These situations suggest that trusting and being able to read equine behavior are factors favored above and potentially *instead of* wearing a safety helmet, although a rider’s ability to read behavior was taken for granted as ethologically accurate. The attitude that risk could be avoided by other mitigation strategies, above and potentially instead of helmet use, was particularly evident in comments by those who openly declared non-consistent helmet use:

- One rider explained that, “*I don’t usually ride with a helmet at home doing flat work but if I go out, ride an un-educated horse or new horse or do any jumping at all I’ll pop one on*”. Only situations self-categorized as unsafe and unpredictable required a helmet, and this rider later noted that they saw helmet use as dependent on knowing a horse and the level of rider experience.
- Similarly, another rider noted that they usually rode without a helmet, unless riding a “*new or young*” horse.
- One rider stated that they did not wear a helmet while training for dressage but would do so if they were riding a young horse, jumping or riding on the roads.
- While listing a number of situations in which they did wear a helmet, one rider mentioned being *a* “*culprit for not wearing a helmet when schooling dressage at home*”, but only while riding their or their mothers “*very experienced*” horses as they “*know damn well they won’t do anything*”.
- Surprisingly, one rider attributed their head injury not to failure to wear a helmet, but a failure to correctly interpret the horse’s behavior.

When risk was present as uncontrollable and outside of the horse/human dyad, then helmet use was considered necessary:

- An openly helmet resistant rider stated that horse riding is “*dangerous as hell*” but that they were not consistent in wearing a helmet. Despite this, they stressed that “*I will however, always wear one on the road!*”

In these examples, the risks involved in equestrian activity were perceived and understood in varying ways by forum members. Helmet use was seen as a tool to mitigate some risks, but other risk limitation strategies, such as trusting and knowing horses, familiarity with riding situations and locations, relationship between the horse and human and levels of rider and horse experience/expertise were often valued over, and often *instead of,* the use of a safety helmet.

#### 3.2.2. “It Does Not Feel Right…”

While some riders found helmets uncomfortable, others found not wearing a helmet more uncomfortable. In this, the attitude that “It Does Not Feel Right” had an unpredictable and bi-directional affect on helmet use behavior. Further, in some cases the strength of social influence meant that the perceived physical discomfort of helmets did not always prevent use.

**“…to wear a helmet”**

A few riders commented that wearing a helmet did not feel right. In some cases, this attitude resulted in non-use, but in others helmets were still worn:

- Justifying their general lack of helmet use, one rider noted “*My usual place of residence is in the tropics and I prefer to wear something that shades my face etc. and allows for airflow*”.
- Similarly, another exclaimed “*All I can say is once you try riding without a helmet it is really hard to go back!*” After training in an environment that non-wearing was encouraged they stated to “*much more able to concentrate without that heat, sweat and constriction*”.

In some cases, the discomfort of wearing a helmet was not a barrier to use, as other factors that encouraged the use of helmets had more behavioral impact:

- One rider commented that they did not grow up wearing helmets, “*hates*” them as they are hot and uncomfortable, but started to wear once their kids started riding as you “*can’t really expect them to wear one if your not*”. For this rider, being a positive role model for their family was valued more than the perceived negative sensation of helmet use.
- Another rider stated simply that they “*always have and always will wear a helmet*” despite hating the red lines they leave on their forehead.
- Despite the negative sensation associated with helmet use, another associated the decision to wear with wanting to be around to see their grandchildren.
- Another stated that they never wore a helmet when younger but had got used to wearing one after the rules changed at their agistment/livery to mandate helmet use and despite still hating them they would feel odd getting onto their horse without one. Though, they noted that they would still wear an Akubra (an iconic Australian cowboy style hat) on long rides for sun protection.

**“…to not wear a helmet”**

While some equestrians viewed the helmet as hot and uncomfortable, presenting a barrier to use (although, as seen, not always), our findings suggest that the physicality of the helmet was not always seen as a negative:

- Wearing a helmet was likened on a number of occasions to putting on clothes everyday, with one rider stating they would feel “*naked*” without their helmet, and another noting that “*not wearing a helmet to me feels like not wearing a bra*”.
- For three riders, wearing a helmet was such a habit that they joked they felt weird not wearing it in other “risky” situations such as driving a car.
- One rider noted wearing their helmet added to their comfort levels as it warmed their head on cold days.
- Three riders simply stated that wearing a helmet was “*second nature*” and not wearing feels “*odd*”.

These findings suggest that helmet use was not always considered uncomfortable, that discomfort was not always a barrier to use and helmet wearing had the potential to become a physically comforting habit, enabling use.

#### 3.2.3. “Accidents Happen”

Similar to the attitude “I Can Control Risk”, those expressing the attitude that “Accidents Happen” perceived risk on a scale, and valued a range of risk mitigation strategies. However, for a majority of those who held this attitude, nothing negated the need to wear a safety helmet. Forum members with this attitude stressed that “accidents happened”, even to the most experienced riders, and that a helmet provides an easy method of injury prevention in these random circumstances:

- One rider noted that in their experience, the only injury incidents they had experienced were from “*accidents*” such as the horse tripping.
- Another stated that wearing a helmet is a “*very basic*” precaution that could change the severity of injury in an accident event.
- Another noted wearing a helmet on their “*quiet horse*” during equestrian sports as it meant they could focus on their/their horse’s performance instead of worrying that the “*unexpected*” might happen.
- One stressed that they always wore a helmet, as “*even ‘bomb proof’ horses can trip. A friend of mine broke her neck riding a bomb proof school horse that tripped in the paddock at home (she is a professional rider)*”.
- The view that even quiet and trusted horses can fault and experienced riders can have accidents was also held by another rider who noted a few occasions where a helmet had saved them in an accident with a quiet, known and trusted horse.
- One rider told the story of a professional dressage rider who had sustained an injury to stress that “*ANYONE can come off, no one is invincible*”.
- Another shared a similar story of a fatal accident in which a very experienced rider fell off, stating, “*experienced people do fall off too*”.

The attitude of “Accidents Happen” provided protection for some riders against the association of experience with a reduced need to wear a helmet. It allowed these riders to present themselves as experienced equestrians while still choosing to wear a helmet.

The attitude that experience negates the need for helmet use is challenged by (self-stated) experienced riders through tales of “accidents that really did happen”. Here, understanding the importance of wearing a helmet had come about through the personal experience of being involved in, or knowing of someone involved in, an accident:

- While admitting not liking helmets when younger one rider stated that they would never “*dream of getting on a horse without one these days*”, sharing the story of being involved in an accident where they had luckily decided to wear a helmet which had lessened the severity of injury.
- Similarly, another highlighted that after witnessing a horrific accident of a non-helmeted rider, they “*would not consider wearing a helmet these days*” despite previously being a bit “*hit and miss*”.
- Another equestrian wrote that they have never ridden without a helmet since being in an accident as “*I shudder when I think back that I had tossed up whether I should wear the helmet before I got on him that fateful day*”.

Riders such as those represented above demonstrated a positive attitude towards helmet use and injury mitigation; they valued risk limitation strategies such as knowing and trusting horses, rider experience and knowing how to read equine behavior *on top of* the added safety measure of safety helmet use. For this group, safety helmets were stated as consistently used because, despite best intentions, “Accidents Happen”.

## 4. Discussion

Through discourse analysis of discussions on two Australian based public-access online forums we found that helmet use was intrinsically linked to the social worlds of equestrians, despite often being viewed as a “personal choice”. Further, we found two attitudes that demonstrated a bidirectional affect on helmet use: “I Can Control Risk” and “It Does Not Feel Right…” and one that enabled helmet use: “Accidents Happen”. We have summarized these attitudes in the table below.

**Table 1:** Barriers and enablers to helmet usage.

Attitude	Encourages Helmet Use When	Discourages Helmet Use When
“I Can Control Risk”	Helmet use seen as part of risk control	Helmet use seen as extemporaneous to other controls such as being a good rider, having a good horse, and having a good relationship with that horse
“It Does Not Feel Right…”	Wearing a helmet becomes a habitual sensation of riding, even if that sensation is discomfort	Wearing a helmet is considered intolerable
Accidents Happen	Accidents accepted as beyond control, and unrelated to rider skill, horse temperament or the quality of their human–horse relationship	The rider has a fatalistic risk perception

The social worlds of the equestrian cohort had a significant influence on helmet use attitudes and behaviors. Attitudes and behaviors towards helmet use were overtly influenced by perceived social responsibility and “care” as well as social regulation in particular riding contexts for some riders, and more subtly formed through social influence and behavioral norms for others. The latter point is consistent with previous research that has demonstrated the negative influence of peer pressure and cultural norms of particular riding styles on helmet use [27,28,29]. The influence of riding peers as a barrier to helmet use was exemplified by one rider who noted that “*I spent a few months training with a well-known show jumper in NSW, basically nobody wears a helmet there and I just got used to it*”. Yet, peer behavior and perceived social responsibility also supported helmet use. For example, one rider noted that wearing a helmet is something one does for “*those who love you*” and another wore a helmet particularly to be a good role for their children, as you “*can’t really expect them to wear one if you’re not*”. This strength could be targeted in injury prevention campaigns. Indeed, research specifically into the adoption of other types of safety helmets has highlighted the significant positive influence peer behavior and social norms could have on consistent use. In an Austrian study, it was found that the positive effect on youth ski helmet adoption that mandatory youth ski helmet laws had in one province was mirrored in another province without mandatory laws [48]. This was attributed to the high ski helmet use by adults and parental figures, which acted to model appropriate and safe behavior [48]. The potential of adults acting as behavioral role models towards helmet use was seen as well in an observational study of bicycle riders in the USA [49], which found that helmet use was higher in pairs of cyclists than in solo riders, and particularly child adult pairs where the adult wore a helmet [49], supporting the strength of companionship as a potential enabler in the promotion of public health interventions. The suggestion of using role modeling to promote desired safety behaviors is stressed in a US study of factors affecting children’s bicycle helmet use which found that regardless of the presence or absence of mandatory laws, use of helmet was associated with beliefs of social consequences and peer use [50]. The researchers suggest communication targeting the social desirability of helmets alongside helmet use by parents to model appropriate behavior for children [50].

The adoption of safety helmets by those in equestrian “role model” positions could also act to break down the association between helmet use and inexperience. Moves by prominent horse media magazines or websites (such as *Dressage Today*) to only publish images of helmeted riders are excellent initiatives in this regard. In this, we suggest investigating and trialing avenues for the promotion of helmet use as a normalized and social activity, no matter what the level of perceived risk, or ability to mitigate risks through skill is held by the rider. This might include petitioning for widespread horse media adoption of helmet only image publication policy as well as injury prevention campaigns that leverage off the helmet use enabler presented by those riders who saw helmet wearing as an action of responsible horse owners and family/community members.

The attitude “I can control risk” demonstrated that the risks involved in equestrian activities were perceived and understood in varying ways. Positively, helmet use was seen as a tool to mitigate risks, but worryingly other risk mitigation strategies were often valued over and above the use of safety helmets. This finding is consistent with that of Reed and colleagues [28] and Condie and colleagues [29], who identified differing risk perceptions in different equestrian contexts as potential barriers to the use of helmets. Moreover, the relationship between risk perception and prevention is not straightforward. Some riders may (more or less consciously) not want to acknowledge risk by taking precaution. 

Further, the construction of horse riding, and horses in general as dangerous and unpredictable animals could unintentionally reduce the energy that riders invest in building a “strong” relationship with their horses [40,51,52]. Challenging this relationship might prove difficult, especially as riders rarely described their own horses as dangerous or unpredictable. Knowing a horse, and being able to read equine behavior was favored strongly as a risk mitigation strategy as part of the attitude that “I can control risk”. This was exemplified in the comment by one equestrian that they ride their family’s “*very experienced*” horses without helmets, as they “*know damn well they won’t do anything*”.

In a similar light, our findings demonstrated some of the factors contributing to risk (mis)perception were possibly inflated self-perceptions of the equine knowledge, skills and experience that are preventative factors for equestrian injuries and have positive consequences for equine welfare [53,54,55,56]. There is a particular challenge in removing any stigma attached to helmet use that riders may interpret as an indicator of a rider’s poor equestrian skills, a horse’s bad temperament or their weak horse-rider relationship. However, our research suggests risk-reduction value in injury prevention campaigns that leverage off the attitude “Accidents Happen” and also normalize helmet use by increasing usage amongst equestrian role models. There may also be value in creating an association between helmet use and being a “good carer” for the horses that depend on riders [57], and an equestrian sector that could be jeopardized by increasing injury statistics.

The finding that physical sensation, comfort and habit influenced helmet use behavior is consistent with Condie and colleagues’ [29] study that found that the perception of helmets as uncomfortable presented a barrier to use. This was also highlighted in studies of English-style riders [58], farm youth [28] and roughstock rodeo athletes [27]. Yet, while the perception that a helmet is uncomfortable and “It Does Not Feel Right” was similarly observed in this data, some riders expressed an opposing positive attitude of “It Does Not Feel Right…to not wear my helmet”. This suggests that the sensation of helmet wearing can be viewed positively and has been effectively normalized by some riders. Further, even when wearing helmets was seen as uncomfortable, this did not present a barrier for some riders, as other values and attitudes exerted more influence on the decision to wear, Particularly strong was the desiring to be a good role model or wanting to “*be around*” for family. This shows again the advantage of utilizing and promoting valued social roles to encourage use and as a result normalize the helmet wearing sensation. The view of equestrians who noted that wearing a helmet felt normal demonstrates there is potential for supporting the idea that helmets can become “*second nature*” and that not wearing can also become an uncomfortable sensation. Indeed, several horse riders likened not wearing their helmet to being uncomfortably “*naked*”. While the physical sensation of helmets has previously been identified as a barrier to use, our analysis suggests that this is not always the case. There is need for current phenomenological and ergonomic research into the physicality of helmet wearing. Promotion of values that override the negative side of the attitude of “It Does Not Feel Right”, particularly those focused on the social would help contribute towards consistent helmet use. Supporting and promoting the attitude that “It Does Not Feel Right… to not wear my helmet” could further work towards normalizing, for a larger number of equestrians, the sensation of helmet wearing.

Interestingly, some forum members displayed attitudes of “Accidents Happen” *and* “I Can Control Risk”. Whilst most saw the use of helmets as not negotiable, there were a few for whom the attitude “Accidents Happen” was interpreted fatalistically to justify not wearing a helmet. Overall, the “Accidents Happen” attitude was linked with the use of safety helmets. It also provided protection for riders against the association between helmet use and inexperienced suggested in the attitude of “I Can Control Risk”. By maintaining the attitude that “Accidents Happen”, riders could present the idea that their experience did not detract from the need (or “choice”) to wear a helmet. Supporting and promoting this attitude could help to alter the inconsistent helmet use of those who predominantly believe “I Can Control Risk” but without threatening the positive injury mitigation strategies that this attitude also contains. Harnessing this attitude could encourage holistic equestrian safety habits that understand the importance of injury countermeasures such as the development of skills and confidence in equine interaction but *alongside* the adoption of an Australian safety standard helmet.

In conclusion, our findings suggest that there is unharnessed potential in injury prevention approaches that strengthen the enablers of equestrian helmet use. In this regard our research extends previous studies of voluntary use of safety equipment in activities such as cycling [31,33,34,49,59], motorcycling [35,36] and winter sports [38,39,48]. Previous studies on equestrian helmet use provide a valuable illustration of the range of attitudes, beliefs and factors that result in helmet non-use [27,28,29,58]. Yet, through accessing naturalistic helmet use discussions between equestrians, we found that many of these factors can also support helmet use. We also highlighted the added sensitivity required in the promotion of equestrian safety equipment, due to the complexity of the horse and human relationship in not only creating [25], but also mitigating, risks [40]. We have illustrated an unexplored and unrealized inroad for equestrian injury prevention interventions by shifting the predominant research focus away from barriers to use, towards working with already existing strengths and values held by “horse people” [60] that support and promote voluntary helmet use [61]. Importantly, we identified an enabling attitude that “Accidents Happen”. This attitude contained a holistic approach to injury prevention which valued increasing equestrian skills, experience, confidence and positive horse/human interaction *alongside* the consistent use of safety helmets because, despite best intentions, “Accidents Happen”. The findings suggest that values important to equestrians already have the potential to assist mitigate risk. These included the importance of a “strong” human and horse relationship, the influence of local “eque-cultural” communities, the importance of acquiring knowledge of equine behavior as well as the impact of comfort and physical sensation of riding. Injury prevention interventions might benefit from maintaining these important values, and strengthening their influence on the desired behavior of helmet use.

## 5. Limitations and Further Research

The qualitative depth of this study exempts the findings from generalizability, but offers a unique glimpse into the “everyday talk” [42] of equestrians about helmet use, providing a range of avenues for further research. Whilst not immediately evident in the analytic phase, the data may have been affected by differences in the target audiences of each forum (*i.e*., junior and adult, modern and traditional riding styles, ‘English’ and ‘Western’ styles). Due to the online sampling, the lack of demographics available about the authors of forum comments restricted larger conclusions that could link types of riders, form of riding discipline, level of experience or population subgroups to particular helmet use attitudes. Given that a rider’s ability to interpret horse behavior and body language could increase the predictability of horses [62], it could also be beneficial to compare self-reported skills with objectively evaluated skills. Such research should also determine the impact of high correlations on helmet use, as an ability to correctly read and predict horse behavior is more likely to reduce the likelihood of an injury more than its consequence. As with any piece of research, opinions expressed in the forums may have subsequently changed. However, there have been no major environmental or legal changes in Australia since 2004 that are considered to have significantly impacted helmet use or attitudes.

To determine the generalizability of these attitudes arising from a particular group of people using a particular forum, further research could utilize surveys, interviews or focus groups with equestrians. Doing so would provide the added bonus of gathering demographic information of riders, which would add to a more detailed analysis of helmet use attitudes. Further research could also involve quantitative research incorporating validated psychometric surveys of sensation seeking [63], as well as evaluating self-efficacy or the physical and psychological impact of previous injuries on helmet use. Focus groups could be particularly beneficial in relation to developing behavior change initiatives and trialing public health messaging and communications. They would be particularly suited to evaluating interventions leveraging from the attitude that “Accidents Happen” and the social implications for individual helmet use decisions on the horses under riders’ care, their families and the broader equestrian community. Similarly, surveys and/or observational research methods could also be useful to extend our understanding of the social influences on helmet use by considering using role models to promote voluntary helmet use. Importantly, our research has shown that shifting the research focus away from helmet use barriers has allowed the identification of already existing helmet use enablers. As a result, behavior change may be acheivable not by challenging long-held and stubborn individual beliefs, but by redirecting them into precautionary behaviors that benefit the whole community.
